# MINARO DRS: usability study of a robotic-assisted laminectomy

**DOI:** 10.1007/s11548-024-03285-x

**Published:** 2024-11-09

**Authors:** Manuel Vossel, Lukas Theisgen, Noah Wickel, Lovis Phlippen, Rastislav Pjontek, Sergey Drobinsky, Hans Clusmann, Klaus Radermacher, Christian Blume, Matías de la Fuente

**Affiliations:** 1 Chair of Medical Engineering, Helmholtz Institute for Biomedical Engineering, RWTH Aachen University, Pauwelsstraße 20, 52074 Aachen, Germany; 2 Neurosurgery Clinic, Uniklinik RWTH Aachen, Pauwelsstraße 30, 52074 Aachen, Germany

**Keywords:** Robot, Robotic-assisted, Cooperative, Synergistic, Hands-on, Laminectomy, Spine surgery, Usability study, Neurosurgery

## Abstract

**Purpose:**

Although the literature shows that robotic assistance can support the surgeon, robotic systems are not widely spread in clinics. They often incorporate large robotic arms adopted from the manufacturing industry, imposing safety hazards when in contact with the patient or surgical staff. We approached this limitation with a modular dual robot consisting of an ultra-lightweight carrier robot for rough prepositioning and small, highly dynamic, application-specific, interchangeable tooling robots.

**Methods:**

A formative usability study with N = 10 neurosurgeons was conducted using a prototype of a novel tooling robot for laminectomy to evaluate the system’s usability. The participants were asked to perform three experiments using the robotic system: (1) prepositioning with the carrier robot and milling into (2) a block phantom as well as (3) a spine model.

**Results:**

All neurosurgeons could perform a simulated laminectomy on a spine phantom using the robotic system. On average, they rated the usability of this first prototype already between good and excellent (SUS-Score above 75%). Eight out of the ten participants preferred robotic-assisted milling over manual milling. For prepositioning, the developed haptic guidance showed significantly higher effectiveness and efficiency than visual navigation.

**Conclusion:**

The proposed dual robot system showed the potential to increase safety in the operating room because of the synergistic hands-on control and the ultra-lightweight design of the carrier robot. The modular design allows for easy adaptation to various surgical procedures. However, improvements are needed in the ergonomics of the tooling robot and the complexity of the virtual fixtures. The cooperative dual robot system can subsequently be tested in a cadaver laboratory and in vivo on animals.

## Introduction

Machining bones is integral to numerous surgical disciplines. It involves procedures such as opening the skull during a craniotomy, inserting screws for spinal fusion, and creating cavities for joint implants. Even though no final scientific agreement is yet established on whether robotic-assisted surgery is superior to conventional surgery [1, 2], for several fields, meta-analyses tend toward an advantage of robotic assistance (RA), especially regarding enhanced accuracy and smaller incisions [3–6]. However, while RA potentially enhances the accuracy of many bone machining surgeries, using robots in the operating room (OR) has introduced new challenges [2].

Current robotic systems employ various kinematic approaches, ranging from large cart-mounted serial arms to miniature, parallel kinematics, either bone-mounted or handheld. Most manufacturers offer large and stiff robotic arms to execute delicate surgical tasks. These medical robots are comparable to industrial robots adapted to meet the operating room’s unique needs [7]. Owing to their substantial mass and long levers, powerful actuators must be integrated into every joint of the robot. These powerful actuators, combined with the oversized workspace, pose potential risks to patients and surgical staff during the robot’s movement [8, 9]. Small application-specific kinematics have been developed to overcome these issues. They can be mounted directly onto the patient’s bone [10]. Advantageously, such kinematics decrease spatial footprint and workspace while improving safety for the surgical staff [10]. Small, handheld robotic systems, in contrast, do not require a rigid fixation to the patient’s bone [11–13]. However, the system’s reduced stiffness and surgeon-induced tremor are challenging. These factors reduce positioning accuracy compared to robotic arms, even when introducing motion compensation [11, 14].

Furthermore, robotic systems differ in their interaction with surgeons [15], varying from semi-active and synergistic to fully active. Synergistic systems can be divided into three modes: handheld [16], hands-on [5], and telemanipulated [17], each featuring characteristic advantages and disadvantages [15]. Synergistic systems combine the robots’ high geometric accuracy and efficiency with the human’s ability to process complex stimuli and react to unforeseen events.

Despite the variety of kinematic approaches and interaction modes with surgeons, nearly all currently available robots for bone machining share one common characteristic: They are restricted to a minimal number of surgical procedures or even a single application [18, 19]. However, their high acquisition costs hinder the widespread adoption of these robotic systems [20–22]. Apart from the limited coverage of applications, most robots currently used are stiff serial arms [23] that are “still too bulky” [24], cause several safety hazards [8, 9], and need to be slowed down so that the supervising operator is able to interrupt the movement at any moment [25, 26].

**Fig. 1:**
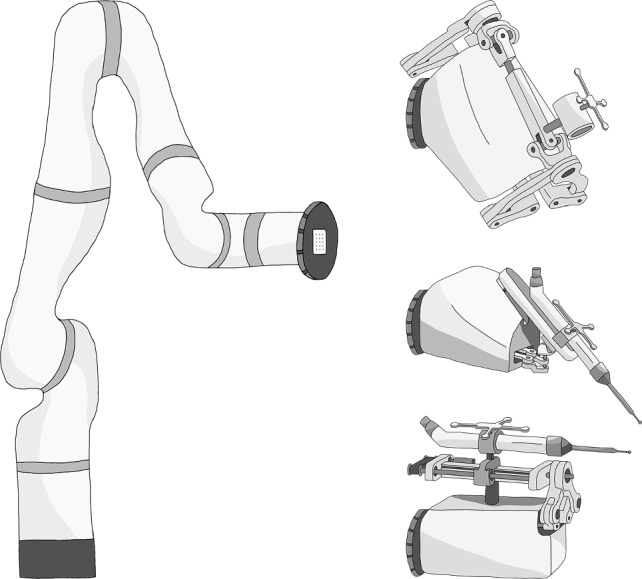
Sketch of the cooperative dual robot system with the carrier robot on the left and three different tooling robots on the right. From top to bottom: Tooling robot with a guide for drilling tasks, milling robot with a rather cubic workspace ideal for laminectomy, and milling robot with a cylindrical workspace for unicompartmental knee arthroplasty (UKA)

We addressed these limitations of powerful and stiff robotic arms used for only a few applications by developing the MINARO Dual Robot System (DRS). The system is dual in the sense that it combines two robots: (1) a large robotic arm and (2) small kinematics. The robotic arm serves as the carrier robot, to which interchangeable tooling robots are mounted (Fig. 1). The carrier robot performs coarse prepositioning, moving the tooling robot into the surgical field of the planned procedure. The tooling robot then executes the surgical plan. This tooling robot is designed to be highly dynamic, quickly adapting the tool position and correcting any elastic deformation of the robotic system. As a result, the carrier robot does not need to be overly stiff, allowing the use of an ultra-lightweight robotic arm with reduced motor torques. This slender carrier robot saves space in the already crowded OR and reduces the risks posed by powerful robotic arms, as described above.

This paper covers the first formative usability study of the MINARO DRS in compliance with IEC62366-1. Ten neurosurgeons evaluated (1) the usability of the prepositioning task, that is, the surgeon steering the carrier robot with the assistance of haptic guidance or visual navigation and (2) the achievable accuracy and general usability of a simulated surgical task using hands-on synergistic control.

## System and controller description

**Fig. 2:**
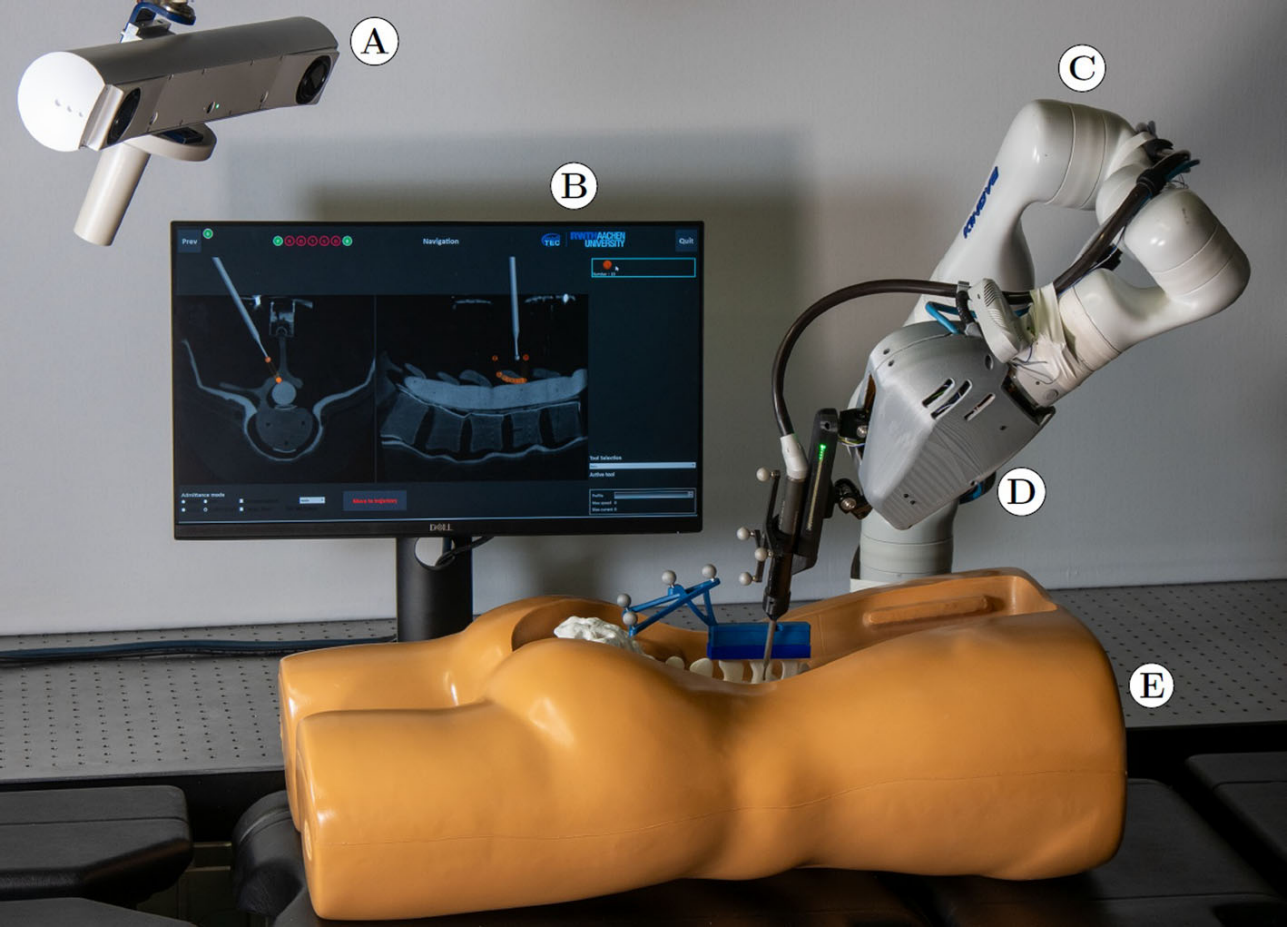
The Minaro DRS with an optical tracking camera **A**, a monitor with the planning and navigation software **B**, the carrier robot **C** with the attached tooling robot (D), and a trunk with spine phantom **E**

The MINARO DRS comprises a carrier robot and interchangeable tooling robots, a tracking camera, a robot control unit (RCU), and a planning and navigation station (Fig. 2). For the tooling robots, we developed motion compensation control to compensate for any patient movement or robot elasticity. Additionally, we developed synergistic control for the hands-on movement of both robots with haptic guidance. All components and control algorithms are briefly described.

### Components

The carrier robot is a Gen3 7 DoF (Kinova Inc., Boisbriand, QC, Canada), one of the most slender and elastic off-the-shelf robotic arms. It provides a maximum reach of 902 mm, a continuous payload of 4 kg mid-range, and weighs just 8.2 kg. The low weight and, therefore, less powerful actuators increase the safety in the operating room. At the same time, the payload and reach are sufficient to position the tooling robot anywhere along the patient’s spine. Using the seventh degree of freedom, the carrier robot’s elbow can be freely positioned not to hinder the surgical staff.

The tooling robots are mounted on the distal end of the carrier robot and hold either the power tool the surgeon works with or a guide for a power tool. The forces the user implies to the system are measured to enable synergistic hands-on control. Therefore, a force/torque sensor (FTD-TW-MINI45-ERA-1,8 SI-290–10, ATI Industrial Automation, Apex, NC, USA) is installed between the carrier and the tooling robot. For the first prototype of the MINARO DRS, we developed a tooling robot for laminectomy. This tooling robot can move a surgical milling instrument in three degrees of freedom inside an imaginary cube with a 43 mm edge length. This workspace is sufficient to remove one entire lamina [27] while simultaneously compensating for the vertebra’s motion due to ventilation [28] and robot deflection [29].

The carrier and tooling robots are draped separately (not shown in the pictures) to maintain sterility during an intraoperative exchange of the tooling robot. For instance, during spinal decompression surgery, one tooling robot is dedicated to milling in the lamina while the other assists with pedicle screw placement. A sterile interface between both robots fixates the tooling robot and provides an electrical connection [30].

A surgical power tool (Anspach eG1 High Speed Electric System, DePuy Synthes, Raynham, MA, USA) with a 4-mm fluted ball burr (L-4B-G1, DePuy Synthes) is clamped to the tooling robot’s end effector, outside the drape. The user activates the power tool by pressing a ring-shaped button integrated into the tooling robot’s clamp that encloses the power tool. The tracking camera is a fusionTrack 500 (Atracsys LLC, Puidoux, Switzerland), being able to track multiple passive arrays simultaneously at 335 Hz. The planning and navigation software runs on a Windows workstation.

The RCU is a tower workstation with Ubuntu Linux and the PREEMPT-RT patch running on it. The force/torque sensor is connected to the RCU via serial RS-232. The other hardware components are connected via Ethernet cable, with the tracking camera using UDP/IP, the tooling robot using EtherCAT, and the carrier robot and the planning and navigation workstation using a mixture of UDP/IP and TPC/IP. The RCU runs the software that integrates all hardware components and runs the control. A separate process is started for each hardware and control element, and the inter-process communication is realized using the Ach library [31].

### Motion compensation control

The dynamic motion compensation control must ensure accurate positioning of the surgical instrument. This compensation does not only include position tracking of the patient to compensate for any movement of the bone. External forces on the robots lead to elastic deflection, which must also be compensated for. Imprecision in the manufactured robot also results in a different tool position than intended, which the dynamic compensation corrects.

The dynamic motion compensation must not excite the robotic system at any resonant frequency through the acceleration of masses moved in the tooling robot. Next to this self-excitation, external excitation through the milling process can occur. Any control instability and, thus, oscillation of the tool must be prevented. Therefore, a filter was added to the control loop to dampen signals starting at the robotic system’s lowest resonant frequency.

In previous experiments, we determined that the system’s lowest resonant frequency highly depends on the carrier robot’s joint configuration, ranging from 4 to 10 Hz. We modeled the robotic system as a simple, one-dimensional damped harmonic oscillator with a fixed mass and fixed damping ratio and a spring constant depending on the carrier robot’s joint configuration. This spring constant is taken from an elastostatic model of the carrier robot that we designed [29]. With this model, we can estimate the lowest resonant frequency with an accuracy of 10% and adapt a second-order band-stop filter accordingly during surgery.

### Synergistic control

Autonomous movement of the carrier robot inside its large workspace would pose a risk to the patient. Therefore, we chose hands-on synergistic control for the carrier robot’s prepositioning task. The surgeon steers the carrier robot while grasping it, ensuring that the surgeon is fully aware of the robot’s movement. Without anyone grasping the carrier robot, it will not move. Haptic guidance is integrated into the robotic system via virtual fixtures (VFs) to assist the surgeon during prepositioning [32, 33]. For the small tooling robot, several control modes are possible, ranging from autonomous movement to hands-on synergistic control and telemanipulation. A modular approach was followed to enable a flexible selection of the mode of operation for different surgical tasks [15, 34].

For the experiments described here, only hands-on synergistic control was used. Both robots of the MINARO DRS possess non-backdrivable kinematics. Therefore, we chose admittance control [35] to implement this hands-on mode of the robotic system. The admittance control acts anisotropically through integrated VFs to add haptic guidance by the robotic system [33]. Motions that the computer system considers helpful, for example, to reach a specific target position, are supported. In contrast, motions in opposite directions are harder to perform or blocked [36].

VFs are often divided into guidance virtual fixtures (GVFs) and forbidden region virtual fixtures (FRVFs) [37]. GVFs guide the operator toward a point or along a path or surface. FRVFs are used to prevent the operator from entering a region. During the prepositioning step with the carrier robot, GVFs aid the surgeon in aligning the carrier robot with the target position for the milling tool at the patient’s lamina. After the target is reached during the prepositioning step, FRVFs are activated to lock the target position in place. Only the remaining degrees of freedom can be changed, that is, rotating the tooling robot around the target position.

During the milling step with the tooling robot, FRVFs are used to keep the milling tool within a preplanned, two-dimensional area. This area is defined through a polygon outlining the ventral contour of the lamina and with its dorsal edge being shifted outside the vertebra as an access area. The milling task is performed hands-on, although telemanipulated control and autonomous execution would be possible with the MINARO DRS.

The milling at the inner cortex of the lamina requires a high accuracy of roughly 300 µm, as this is the thickness of the dura mater underneath the laminae [38]. According to the literature, autonomous milling cannot achieve this accuracy. Autonomous milling is limited to the accuracy of image acquisition, planning, registration, and optical tracking, which is worse than the required 300 µm [39, 40]. Since the surgical instruments must not break the dura by any means, current robotic approaches to laminectomy or craniotomy aim to leave bone remnants [41], which then have to be removed manually [42].

The tooling robot of the MINARO DRS follows a different approach: the hands-on synergistic control is enhanced by an iterative stepwise approach (ISA) to benefit from RA beyond the limitations of medical image acquisition. In the first step, the previously planned polygon is shrunk by the maximum error the system is expected to have. The surgeon can then freely move the milling tool in this area. Milling outside of it is not possible. After cutting through the bone up to this safety barrier, the VF can be expanded in small steps. With each step, the surgeon can visually check for bone remnants and move the milling tool to these spots. The VF is only expanded in small steps so the surgeon cannot steer the tool too deep. This expansion must be repeated until the cut is complete.

## Evaluation method

Three experiments were conducted to evaluate the usability of the prepositioning and the milling task in accordance with IEC 62366–1 regarding effectiveness, efficiency, and user satisfaction ([43], sec. 3.16).

The first experiment was a prepositioning task applied to a spine model with the carrier robot. Haptic guidance was tested against visual navigation to compare the usability.

The second experiment was a milling task, conducted on a block phantom. A cut had to be milled into the block, reaching its bottom surface without damaging the foam underneath. The flat bottom surface of the simple block ensured accurate measurement of the depth to which the milling tool penetrated the foam. This experiment evaluated the effectiveness of the tooling robot’s visually monitored, synergistic hands-on control and, therefore, tested whether this exceeds the possible accuracy of automated milling [39].

The final experiment used a spine model to be closer to the final application. The participants were required to cut through two laminae using the robotic system. They performed an additional manual cut comparable to regular surgery. This experiment was conducted to assess the system’s efficiency and user satisfaction.

### Prepositioning

**Fig. 3:**
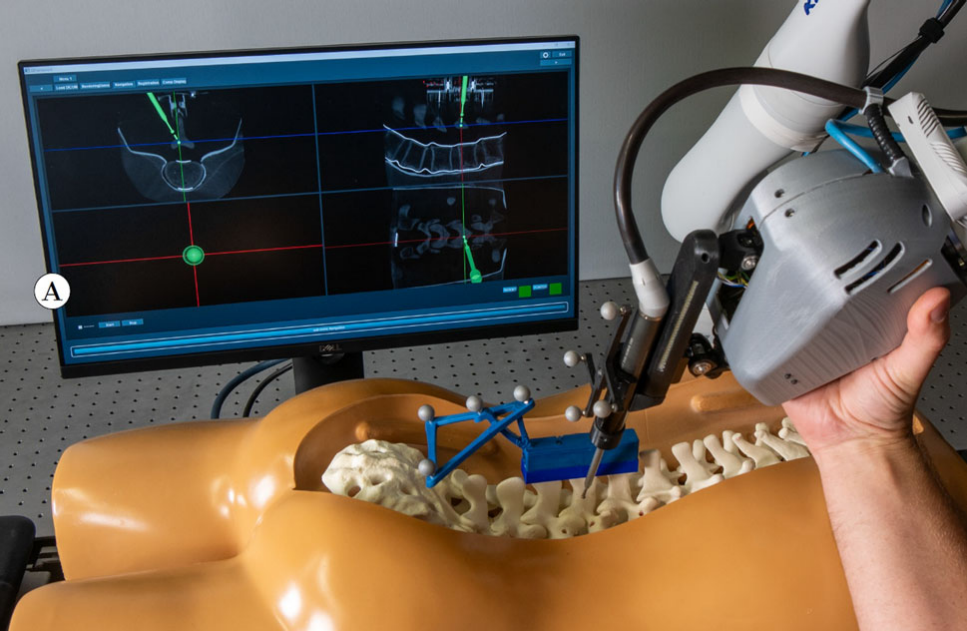
Setup for the prepositioning task: a navigation monitor with a multiplanar view of the CT scan and compensatory display **A**, the user grasping the tooling robot’s housing, and the torso with a spine model

For the prepositioning task, a torso and a spine model (SAWBONES Vertebroplasty Trunk with Spine Model, SKU 1513–19, Pacific Research Laboratories Inc., Vashon, WA, USA) were used (Fig. 3). Prior to this study, a CT scan of the spine model was taken using the Surgivisio system (eCential Robotics, Gière, France). This system automatically performs the registration step to transform the CT coordinates into the coordinates of the optical tracking system.

The participants were divided into two groups to determine which type of assistance to test first. The assignment to the two groups was done alternately. The study supervisor then presented the robotic system to the participants and briefly explained the planned experiments. Participants gave informed consent and completed an initial questionnaire with demographic data, including whether they had previous experience with navigation systems or RA in surgery.

The participants were given instructions for prepositioning with the carrier robot. They could then perform test alignments using visual navigation and haptic guidance. Once they felt comfortable using the system, the experiment began. Each participant performed four prepositioning tasks, two with each type of assistance. During prepositioning with haptic guidance, the navigation monitor was turned off. At the beginning of each prepositioning task, the carrier robot was moved to its starting position.

For visual navigation, the CT scan was displayed in sagittal, coronal, and axial planes, while the current position of the milling tool and the target position were simultaneously indicated. Additionally, a compensatory display showed the relationship between the current and the target tool positions in the coronal plane embodied by a disk and a ring. The size of the disk represented the current position error in the anteroposterior direction. When the participant reached the target by 3 mm, the displayed milling tool switched to green, which was considered a successful task completion.

For haptic guidance, translational GVFs were integrated into the admittance control of the carrier robot. Once the target position was reached, the admittance control switched to FRVF to hold the position, and the LED bar turned green, which was considered a successful completion of the task.

Light rotational GVFs were activated on the carrier robot at all times. Previous tests had shown that aligning the tooling robot to the target without any assistance was challenging, which is consistent with the literature [44]. The rotational GVFs were the same for both assistance types: visual navigation and translational haptic guidance.

With both assistance types, the participants were required to activate the admittance control of the carrier robot by pressing a single button on the tooling robot’s housing. The effectiveness of the prepositioning step was assessed by comparing the milling tool’s optically tracked position when the participant released the button with the target position. Efficiency was determined by measuring the time from the first button press until the final release, as time is one of the most valuable resources during surgery. The usability was evaluated using System Usability Scale (SUS) questionnaires [45] that were completed by the participants after testing each assistance type. An open-ended questionnaire was completed at the end of this experiment. Two-sample t-tests ([46], sec. 31.2) were performed to evaluate whether haptic guidance or visual navigation is significantly better. Significant differences are marked with one, two, or three asterisks denoting p < 0.05, p < 0.01, and p < 0.001, respectively.

### Milling in the block phantom

**Fig. 4:**
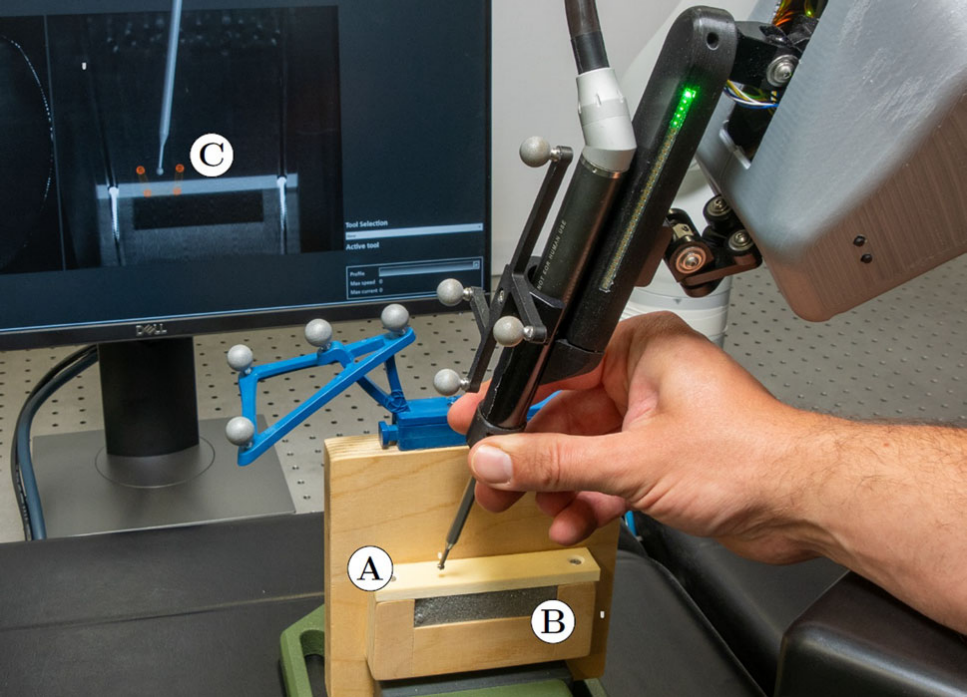
Setup for milling in a block phantom (**A**) with foam (**B**) underneath. The planned polygon (**C**) is displayed on the navigation screen. The user presses the ring-shaped button at the milling tool to activate the tooling robot’s admittance control

The milling workpiece could be reproducibly mounted on a wooden board (Fig. 4). This workpiece was a block of rigid polyurethane (obomodulan® 1200 sahara, OBO-Werke GmbH, Stadthagen, Germany) used as a bone graft substitute. Its volumetric mass density of 1200 kg m^−3^ is close to the density of cortical bone, which is between 1600 kg m^−3^ and 2000 kg m^−3^ [47, 48]. In previous sawing tests, the material demonstrated cutting strength comparable to cortical bone ([49], page 50). The milling material was pressed against a block of very light foam, which offered no noticeable resistance to the milling tool.

Prior to the study, a CT scan of the test bench was taken using the Surgivisio system. Both blocks (milling phantom and foam underneath) could then be exchanged for each participant. In the CT scan, a milling groove was planned as a polygon up to the boundary between the rigid polyurethane and the lightweight foam (Fig. 4C). The maximum registration error between the plan in the image data set and the actual milling tool tip position is 0.59 mm [39]. Adding some safety buffer, the planned polygon (VF of the tooling robot’s admittance control) was reduced by 1 mm for the ISA.

At the start of the experiment, a VF was loaded into the image, which consisted of a polygon placed in front of the block. The participants were then shown how to steer the tooling robot and extend the VF. The participants could familiarize themselves with the system and move the bottom edge of the VF with the ISA. The navigation screen showed the position of the milling tool tip in the CT data set in relation to the planned VF.

Once the participants felt confident in interacting with the tooling robot, the VF was replaced by the polygon inside the block phantom, and the prepositioning was repeated. The participants completely removed the material in this area using the tooling robot with the attached surgical milling tool. After visual inspection, the surgeons expanded the polygon stepwise by 0.1 mm to remove more material in areas with bone remnants. These steps of polygon expansion and removal of bone remnants were repeated until the participants were satisfied with the milling result. During an actual surgery, the surgeons would stop milling at this point because the remaining bone could either be broken away or removed with a Kerrison rongeur according to their experience. During milling, the dynamic compensation control of the tooling robot was turned on and automatically tuned to the estimated resonant frequency of the carrier robot at its current joint configuration.

The milling effectiveness using the tooling robot with the ISA was assessed by measuring the maximum breach through the block phantom. Since a spherical burr was used, the maximum breach could be calculated based on the burr’s radius and the largest circular hole milled through the block phantom.

### Milling in the spine model

**Fig. 5:**
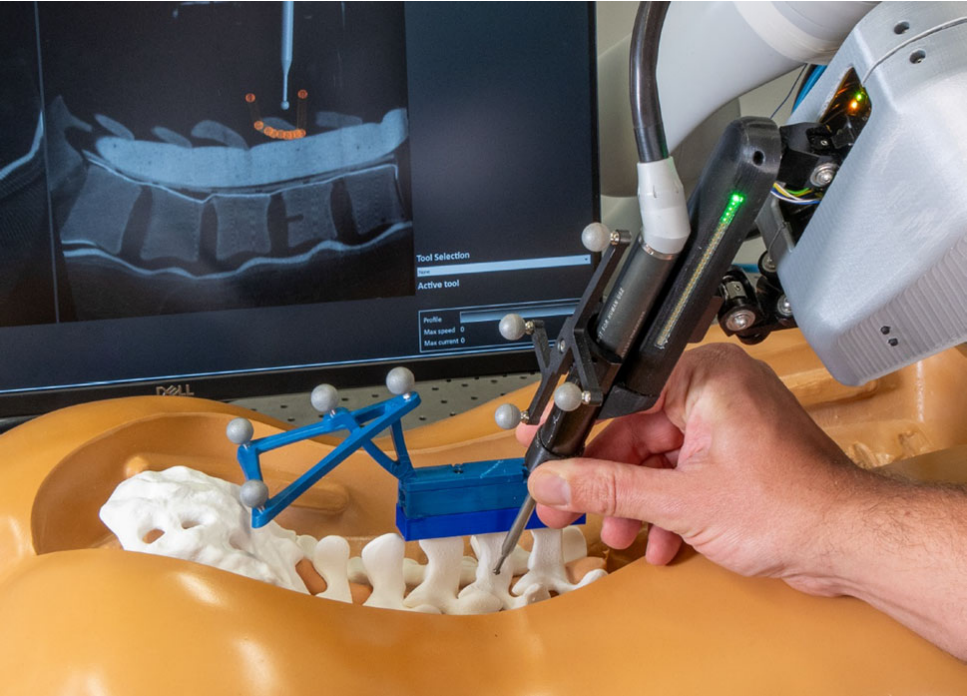
Setup for milling in the spine model

The spine model provides a more realistic laminectomy scenario (Fig. 5). The same trunk was selected as for the prepositioning experiment, but only a lumbar spine model (SAWBONES SKU 1324) was used. The Surgivisio patient reference was fixed to the lumbar vertebrae L1 and L3. As a spinal cord substitute, the spinal canal was filled with a sausage (K-Bio Wiener Würstchen, Kaufland Stiftung & Co. KG, Neckarsulm, Germany). A thin wooden stick was inserted between the sausage and the vertebral body to press the sausage firmly against the lamina.

For each participant, the Surgivisio patient reference unit had to be fixed to a new lumbar spine model. Therefore, a new CT scan was performed for each participant. Planar cuts through the right laminae of L2 and L4 were planned in the image. The participants milled these cuts analogously to the block phantom.

An additional manual cut was performed at the right lamina of L3 to compare the presented spine model to a real patient scenario. Therefore, the participants used a HiLAN surgical milling tool (GD676 microspeed® uni Hi motor, Aesculap AG, Tuttlingen, Germany) with a 6 mm rosen burr (GD 745R, Aesculap AG). A Kerrison rongeur (FKB91B, Aesculap AG) was supplied alongside the milling tool to enable the participants to verify if they had milled sufficient bone substitute material. Afterward, the participants completed another open-ended questionnaire asking for feedback on the tooling robot and the dual robot system in general. The efficiency for all milling tasks was assessed by clocking the participants from the first touch of the burr to the phantom until the participant deemed milling complete. Usability was assessed by evaluating the questionnaires.

## Results

Ten neurosurgeons participated in the formative usability study. The participants were all practicing surgeons at RWTH Aachen University Hospital. Their experience ranged from a novice with only four months of experience in neurosurgery to the head of the department with 29 years of experience at the time of the study. All participants were right-handed, and two were female. All participants had experience with navigation systems, whereas two participants mentioned earlier experience with robotic systems.

### Prepositioning

**Fig. 6:**
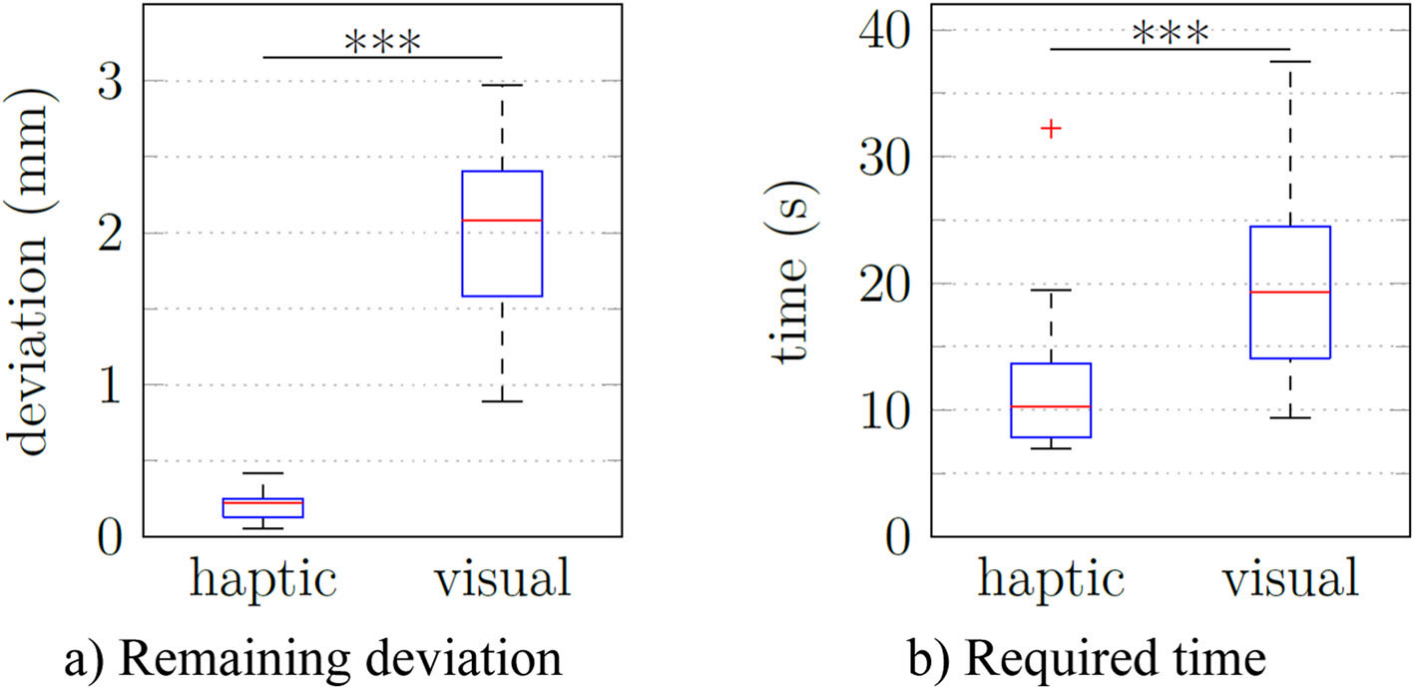
Analysis of the log files recorded during the prepositioning with haptic guidance vs. visual navigation

Regarding the effectiveness of the prepositioning task, the remaining deviation of the tool to the target position was evaluated (Fig. 6a). With haptic guidance, the deviation was (0.21 ± 0.09) mm with a maximum of 0.42 mm. With visual navigation, the deviation was (1.97 ± 0.60) mm with a maximum of 2.97 mm. Both assistance types proved effective, as each participant reached the target each time, but the haptic guidance performed significantly better (p < 0.001).

For efficiency, the time required for the prepositioning was measured (Fig. 6b). With haptic guidance, it took the participants (11.7 ± 5.9) s with a maximum of 32.2 s. With visual navigation, the time required was (19.9 ± 7.8) s with a maximum of 37.5 s. Again, the haptic guidance performed significantly better (p < 0.001).

**Fig. 7:**
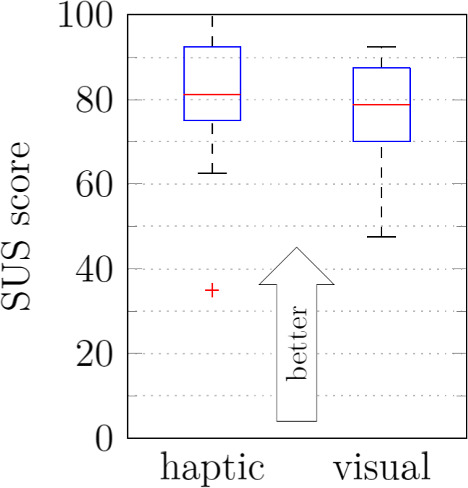
User satisfaction evaluation of the prepositioning with visual navigation vs. haptic guidance

**Fig. 8:**
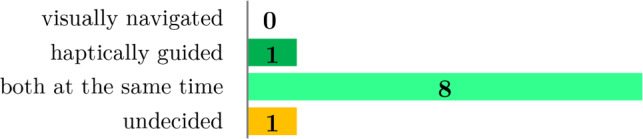
Answers to the question “Which assistance type do you prefer?” after the participants performed the prepositioning experiment

The average SUS score for haptic guidance was 79, while the average visual navigation score was slightly lower at 76 (Fig. 7). No significant difference was found. While completing the open-ended questionnaire, eight participants expressed a preference for using both types of assistance concurrently (Fig. 8). Five of the eight who would prefer both types simultaneously cited difficulty fully trusting machine guidance or a desire for visual feedback on the monitor. Participants noted that the primary advantages of haptic guidance were simplicity (5 out of 10) and speed (6), while the primary benefit of visual guidance was optical control (5).

### Milling

The milling process using the dual robot was considered effective when participants successfully cut through the lamina without damaging the spinal cord underneath, meeting the accuracy requirement of 300 µm, which was evaluated with the experiment on the block phantom. This requirement was reached by seven of the ten participants. One participant once activated the milling tool before engaging the admittance control, potentially prior to fully grasping the tool. As a result, the milling tool was pulled into the material, causing it to penetrate too deep. The other two participants cut between 400 µm and 500 µm into the foam. However, it is unknown whether the participants intentionally wanted to open the cut further.

**Fig. 9:**
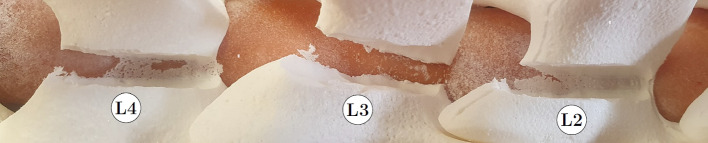
Milling result in the spine model of one participant. L2 and L4 were milled using RA, while L3 was milled manually. The white particles visible on the spinal cord phantom are milling dust. The wide opening of the manually milled cut indicates that the rosen burr must have penetrated the spinal cord

An example of a participant milling in the spine model is shown in Fig. 9. On both RA cuts (L4 and L2), the participant left material at the cranial section (right side in the image), where the spine phantom was pressed against the lamina. A total cut was made at the caudal part. In the experimental laboratory setup, there was an air gap between the caudal part of each lamina and the spinal cord phantom, while in surgery, the ligamentum flavum fills this region.

**Fig. 10:**
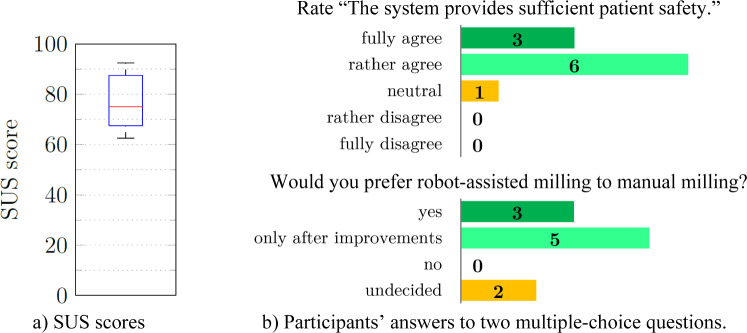
Results of the questionnaires of the milling experiments

All participants reported that the robot facilitated the removal of sufficient material. In surgery, they would then change to different tools, such as a Kerrison rongeur, to remove the remnant bone. After the participants performed all three cuts with RA, they rated the statement about the system providing sufficient safety for the patient (Fig. 10b). The statement received three “fully agree” and six “rather agree” ratings, while one participant remained “neutral.”

Manual milling was significantly faster (p < 0.001) for all participants, except for one, who was the least experienced, who performed RA milling of L2 and L4 faster than manual milling of L3. Manual milling took (65 ± 38) s with a maximum of 167 s, and RA took (141 ± 63) s with a maximum of 258 s. Four participants stated, without being explicitly asked, that manual milling during the actual surgery would take longer because any penetration toward the spinal cord would be more harmful to the patient than to the phantom in this study. During the actual surgery, they would have switched to a diamond burr instead of finishing with the rosen burr. Furthermore, the limited visibility of anatomical structures during actual surgery would make manual milling more difficult than RA milling.

The average SUS score was 77 (Fig. 10a). The participants were asked about their preference for RA milling versus manual milling (Fig. 10b). Three participants favored RA milling. At the same time, five stated they would prefer it “only after improvements.” Two were “undecided,” with one stating that their decision would depend on the individual case. If there were increased thickness of ligaments or other soft tissue between the lamina and dura, the added time required to prepare the robotic system would outweigh the benefits.

The primary improvement sought for the robotic system by the participants was the inclusion of additional surgical plan options to facilitate milling a larger volume, as opposed to just having the current two-dimensional cut. Six participants asked this. The robotic system may prove advantageous for the undercut approach. With this approach, the lamina on one side of the vertebra is cut first. Through this gap, bone material underneath the spinous process and from the other lamina is removed. This approach is more commonly employed than a complete laminectomy, which involves cutting both laminae of one vertebra separately, along with the removal of the entire spinous process. One participant suggested that a more flexible VF planning, along with the use of the robotic system, could pave the way for new surgical techniques that are currently impossible to perform.

## Discussion

The formative study aimed to evaluate the usability of the cooperative dual robot system, including prepositioning with the carrier robot and milling with the tooling robot. Results from all experiments indicated a high user satisfaction rating with average SUS scores ranging from 76 to 79, which is considered “good” to “excellent” [50].

During the prepositioning task, both assistance types, haptic guidance and visual navigation, yielded similarly high user satisfaction. In addition to obtaining favorable user satisfaction ratings, both types of assistance successfully achieved the desired level of accuracy. However, haptic guidance demonstrated higher effectiveness and efficiency. This difference aligns with prior research, which found that haptic guidance can enhance effectiveness and efficiency during teleoperated synergistic control [32].

In terms of the effectiveness of the milling process with ISA, most surgeons were able to meet the accuracy requirement of a maximum 0.3 mm breach while milling the block phantom, which is more accurate than what would be possible with an autonomous approach [39]. It should be noted that this cut in the block phantom was the participants’ first milling experience with the MINARO DRS. The participants had previously tested the interaction with the system by steering the milling tool through and familiarizing themselves with the VF enforcement, while milling was not performed. Therefore, the results were expected to improve after a short training period.

In a laminectomy, as in a craniotomy, it is essential not to cut too deeply into the bone in order to preserve the underlying dura [51]. Current image-based robotic systems for craniotomy or laminectomy typically do not provide the required accuracy to avoid damaging the dura mater while simultaneously completely cutting the bone. Inaccuracies originate from the potential accumulation of errors or uncertainties from imaging, image processing, planning, registration, and tracking [39]. Therefore, these robotic systems leave bone remnants that must be removed manually [41, 51]. The results of this study suggest that the synergistic hands-on control of the tooling robot overcomes the limitations of image acquisition, planning, and registration by making the VFs adaptable during surgery. This could make the MINARO DRS the first robot to allow complete removal of the lamina using only the milling tool attached to the robotic system without any further manual steps.

The efficiency of RA milling was tested by comparing it to manual milling in the spine model. In addition to the two RA cuts, the surgeons performed a manual cut. It was found that, on average, RA milling took twice as long as manual milling. However, the surgeons stated that these measured times could not be transferred to actual surgeries, as manual milling was much simpler on the spine model with an open view of the lamina. Some surgeons also stated that they would be more careful during actual surgery, milling slower toward the spinal cord when an actual patient’s life is at risk, instead of the spine phantom of this study. The time required for RA milling may increase far less in a real setting. As with effectiveness, the surgeons had no previous training with the robotic system despite years of experience in neurosurgery. This hypothesis is further supported by the fact that the surgeon with the least experience in neurosurgery, only four months, was the only one faster using RA than manual milling. Therefore, additional training with the robotic system could decrease the time needed for RA milling. Furthermore, the measured times in this study did not incorporate the use of the Kerrison rongeur. The concept behind the ISA approach was that this manual step might be unnecessary. Examining the fragile layer of material remaining after RA milling (L4 and L2, Fig. 9), which the surgeon noted could easily be broken off during surgery, supports this theory. However, this hypothesis must be verified in a cadaver laboratory or with animal tests rather than relying on this formative study.

Overall, the experiments demonstrate highly encouraging outcomes of the cooperative dual robot concept. Specifically, eight out of the ten participants preferred RA milling over manual milling. Three of those participants even already preferred the robotic system in its current state, without any additional improvements. The primary limitation of this study is the laboratory environment, which utilized artificial phantoms. Tests in a cadaver laboratory with an enhanced tooling robot are crucial for assessing the effectiveness and efficiency of RA milling. The enhanced tooling robot with more extensive software and better overall usability must then also be used to test the system’s learning curve with new participants, as this was not covered in this formative usability study.

The modular approach utilizing application-specific tooling robots has the potential to cover a wide range of surgical procedures. This paper examined one such tooling robot designed for laminectomy, although the surgeons suggested that it could be used for any spinal decompression procedure with only software modifications. Two additional tooling robots were presented (Fig. 1): a milling robot with a unique workspace for UKA and a tooling robot equipped with a guide sleeve for precise drilling tasks.

Although the formative, interaction-centered usability study with ten neurosurgeons performing different tasks with the robotic system suggests the usability of the cooperative MINARO DRS concept, it does not prove that the presented system is superior to existing surgical robots with very rigid kinematics. Further experiments on human cadavers, including hands-on as well as teleoperated milling, will be necessary. However, the modular design of the presented robot is easier to integrate into the clinical working environment than large, rigid robotic arms [52]. This formative usability study has provided a solid basis for future work.
